# Diagnostic Accuracy of Recombinant Immunoglobulin-like Protein A-Based IgM ELISA for the Early Diagnosis of Leptospirosis in the Philippines

**DOI:** 10.1371/journal.pntd.0003879

**Published:** 2015-06-25

**Authors:** Emi Kitashoji, Nobuo Koizumi, Talitha Lea V. Lacuesta, Daisuke Usuda, Maricel R. Ribo, Edith S. Tria, Winston S. Go, Maiko Kojiro, Christopher M. Parry, Efren M. Dimaano, Jose B. Villarama, Makoto Ohnishi, Motoi Suzuki, Koya Ariyoshi

**Affiliations:** 1 Department of Clinical Tropical Medicine, Institute of Tropical Medicine, Nagasaki University Graduate School of Biomedical Science, Sakamoto, Nagasaki, Japan; 2 Department of Bacteriology I, National Institute of Infectious Diseases, Toyama, Shinjuku-ku, Tokyo, Japan; 3 San Lazaro Hospital, Santa Cruz, Manila, Republic of the Philippines; 4 Department of Community Medicine, Kanazawa Medical University Himi Municipal Hospital, Himi City, Toyama, Japan; 5 Department of Infectious Diseases, Nagasaki University Hospital, Sakamoto, Nagasaki, Japan; 6 Department of Global Health, School of Tropical Medicine and Global Health, Nagasaki University, Sakamoto, Nagasaki, Japan; 7 Department of Clinical Research, London School of Hygiene and Tropical Medicine, London, United Kingdom; Universidad Peruana Cayetano Heredia, PERU

## Abstract

**Background:**

Leptospirosis is an important but largely under-recognized public health problem in the tropics. Establishment of highly sensitive and specific laboratory diagnosis is essential to reveal the magnitude of problem and to improve treatment. This study aimed to evaluate the diagnostic accuracy of a recombinant LigA protein based IgM ELISA during outbreaks in the clinical-setting of a highly endemic country.

**Methodology/Principal Findings:**

A prospective study was conducted from October 2011 to September 2013 at a national referral hospital for infectious diseases in Manila, Philippines. Patients who were hospitalized with clinically suspected leptospirosis were enrolled. Plasma and urine were collected on admission and/or at discharge and tested using the LigA-IgM ELISA and a whole cell-based IgM ELISA. Sensitivity and specificity of these tests were evaluated with cases diagnosed by microscopic agglutination test (MAT), culture and LAMP as the composite reference standard and blood bank donors as healthy controls: the mean+3 standard deviation optical density value of healthy controls was used as the cut-off limit (0.062 for the LigA-IgM ELISA and 0.691 for the whole cell-based IgM ELISA). Of 304 patients enrolled in the study, 270 (89.1%) were male and the median age was 30.5 years; 167 (54.9%) were laboratory confirmed. The sensitivity and ROC curve AUC for the LigA-IgM ELISA was significantly greater than the whole cell-based IgM ELISA (69.5% vs. 54.3%, p<0.01; 0.90 vs. 0.82, p<0.01) on admission, but not at discharge. The specificity of LigA-IgM ELISA and whole cell-based IgM ELISA were not significantly different (98% vs. 97%). Among 158 MAT negative patients, 53 and 28 were positive by LigA- and whole cell-based IgM ELISA, respectively; if the laboratory confirmation was re-defined by LigA-IgM ELISA and LAMP, the clinical findings were more characteristic of leptospirosis than the diagnosis based on MAT/culture/LAMP.

**Conclusions/Significance:**

The newly developed LigA-IgM ELISA is more sensitive than the whole cell-based IgM based ELISA. Although the final diagnosis must be validated by more specific tests, LigA-IgM ELISA could be a useful diagnostic test in a real clinical-setting, where diagnosis is needed in the early phase of infection.

## Author Summary

Leptospirosis is an important but largely under-recognized public health problem in the tropics. A sensitive, specific and practical point-of-care laboratory test is needed to improve clinical management in resource-limited settings. We developed a recombinant LigA protein based IgM assay (IgM ELISA) and evaluated the diagnostic accuracy of the test during outbreaks in the clinical-setting of a highly endemic country, the Philippines. Sensitivity and specificity of LigA-IgM ELISA and the whole cell-based IgM ELISA, which is included in WHO guidance, were evaluated with cases diagnosed by the standard serological test, MAT, culture and loop-mediated amplification (LAMP) as the composite reference standard and blood bank donors as healthy controls. The sensitivity and the area under the receiver operating characteristic curve for the LigA-IgM ELISA was significantly greater than those of the whole cell-based IgM ELISA on admission. When we re-defined the laboratory confirmation by LigA-IgM ELISA and LAMP, the clinical findings were more characteristic of leptospirosis than the diagnosis based on MAT/culture/LAMP. Our results suggest that LigA-IgM ELISA could be a useful diagnostic test in a real clinical-setting and help timely initiation of antibiotics to prevent severe outcomes of leptospirosis.

## Introduction

Leptospirosis is a ubiquitous zoonosis caused by over a hundred serovars of the pathogenic spirochetes, *Leptospira* spp. The disease is an important public health problem in low- and middle-income countries in the tropics, especially in Southeast Asia and Latin America, including the Philippines [1–3]. According to the World Health Organization (WHO)-International Leptospirosis Society (ILS), the estimated annual number of leptospirosis cases is 350,000–500,000 [4,5] and annual incidence is estimated from 0.1–1 per 100,000 in temperate climates to 10–100 per 100,000 in the humid tropics [6]. The importance of leptospirosis has been largely under-recognized for several reasons. The disease causes a wide range of clinical manifestations, which mimic other tropical infectious diseases such as malaria, dengue fever, typhoid fever and other hemorrhagic viral diseases [6]. The disease severity also varies from a self-limited non-specific febrile illness to fatal cases. A sensitive, specific and practical point-of-care laboratory test has not been available. The current composite reference standard for diagnosing leptospirosis is culture and microscopic agglutination test (MAT) but these tests are laborious and require a special laboratory facility and skills to interpret results [7]. Establishing a highly sensitive, specific, and practical laboratory-based diagnosis for leptospirosis is essential to reveal the true magnitude of disease burden and to improve clinical management.

Studies have shown that the detection of *Leptospira* DNA using conventional or real time PCR had a higher sensitivity than culture and is useful for early diagnosis [8]. More recently, Loop-mediated isothermal amplification (LAMP)-based method has been developed. This method is fast and more sensitive for testing clinical samples and cost-effective, and more suited in resource-limited settings [9–11]. Limitations of the approach are that bacteremia is transient in the early phase of infection while the spirochetes remain in urine only in a fraction of patients. In addition, in countries where antibiotics are freely available over the counter, patients are often exposed to antibiotics prior to admission, and this affects the detection rate of *Leptospira* DNA.

MAT is the current reference standard serological diagnostic test in leptospirosis, and most studies defined laboratory-confirmed cases based on MAT. MAT method is time-consuming and requires specialist laboratory expertise [12]. As an alternative, whole cell-based serological tests have been developed and used in endemic areas [13–16]. Most of the whole cell-based serological assays employed antigens from non-pathogenic *L*. *biflexa* serovar Patoc, with which sera from patients with leptospirosis cross-reacted, and these assays are believed to be genus-specific and detect IgM antibodies from patients, regardless of infective serovars or serogroups [17]. The disadvantage of the whole cell antigen is that it may cause cross-reaction with other infectious diseases. To overcome this limitation, several recombinant protein-based serological tests have been developed, using outer membrane proteins such as LipL32, LipL41, OmpL1, Loa22 and Lig proteins [18–20]. *Leptospiral* immunoglobulin-like (Lig) proteins are surface-exposed outer membrane proteins, which bind to extracellular matrix proteins and are present only among pathogenic species [21]. LigA protein might be expected to be a good candidate of target antigen for serological tests such as ELISA, and previous studies showed 62.0–92.1% sensitivity in referral human serum samples [10,11,22]. The diagnostic performance of LigA-based ELISA has not been examined in the real-clinical setting of highly endemic area.

In the Philippines leptospirosis is highly endemic. Outbreaks of leptospirosis occur during typhoon seasons particularly in urban slum areas such as Metro Manila [3,6]. In this study, we have evaluated the diagnostic accuracy of a new recombinant LigA antigen based IgM ELISA and compared it with that of the whole cell-based IgM (Patoc-IgM) ELISA in a high-endemic area in the Philippines.

## Materials and Methods

### Patients and samples

A prospective observational study was conducted at a national referral hospital for infectious diseases in Manila, Philippines during three leptospirosis outbreak seasons; October to December in 2011, September to October in 2012 and August to September in 2013. Hospitalized patients were approached for enrollment if they had 1) fever plus at least 2 other signs and symptoms of leptospirosis (headache, myalgia, eye pain, nausea, vomiting, abdominal pain, diarrhea, conjunctival suffusion, jaundice, tea-colored urine, oliguria, anuria, or unusual bleeding) and 2) history of exposure to floodwater or animals [3]. Demographic and clinical data were collected from patients and medical charts using a standardized data collection sheet. Blood samples were taken both on admission (1st sample) and at discharge (2nd sample) or either one of those. Plasma was separated from blood by centrifugation and stored at -20°C. Urine samples were collected on admission and stored at -20°C. As healthy controls, 100 blood donor samples were obtained from the Philippine Blood Center of the Department of Health in Manila. No background information was available for the blood donor samples.

### Sample processing

Two milliliters of the plasma samples were centrifuged at 16,000 × *g* for10 min and the pellets were subjected to DNA extraction using the DNeasy Blood & Tissue Kit (Qiagen) according to the manufacturer’s instructions. Two milliliter of the urine samples was centrifuged at 500 × *g* for 5 min to remove the precipitates, prior to a high-speed centrifugation (16,000 ×*g*, 10 min). The resulting pellets were resuspended in 20 μl of 10 mM Tris–HCl (pH 8.0) containing 1 mM EDTA (TE) and then boiled for 10 min. The supernatant of the boiled sample was used as a template for Lepto-*rrs* LAMP.

### Expression and purification of recombinant LigA/GST fusion protein and GST

The C-terminal portions of LigA (amino acid position 708–1224) fused with GST or GST alone was expressed in *Escherichia coli* BL21 and purified as described previously [23].

### Microscopic agglutination test (MAT)

The MAT was performed for detecting anti-*Leptospira* antibodies in patient serum samples [24] using a battery of the reference strains of serogroups Australis (serovar Australis; strain Akiyami C), Autumnalis (Autumnalis; Akiyami A), Javanica (Poi; Poi), Pomona (Pomona; Pomona), Sejroe (Sejroe; M 84), and Tarassovi (Tarassovi; Perepelitsin) and rat isolates in the Philippines of serogroups Bataviae (Losbanos; K68), Grippotyphosa (unidentified; K93) and Pyrogenes (Manilae; K72), which cover prevalent leptospiral serogroups in the Philippines [3,25]. These strains were cultivated in liquid modified Korthof's medium with 10% rabbit serum at 30°C [24].

### Culture

Culture was performed using Korthof’s medium. After the sample collection, 1–2 drops of blood were put into Korthof’s medium and cultivated at 30°C up to 13 weeks. The cultures were examined weekly by dark-field microscopy. Positive cultures were identified by MAT and PCR.

### Lepto-*rrs* LAMP

Lepto-*rrs* LAMP was performed using previously described primers and conditions [10]. The DNA template was preheated for 5 min at 95°C followed by rapid cooling on ice before addition to LAMP reaction mix.

### Recombinant LigA-based IgM capture ELISA (LigA-IgM ELISA)

The microtiter plates (Immobilizer Amino, Nunc) were coated with anti-human IgM (Jackson ImmunoResearch) at the concentration of 180 ng per well in 50 μl of 100 mM sodium phosphate, pH 8.0 overnight at 4°C. Excess binding sites of the well were blocked with 200 μl per well of 20 mg/ml of BSA in 20 mM Tris, 150 mM NaCl, 0.05% Tween 20, pH 7.5 (TBST) for 1.5 h at room temperature (RT), after which the BSA solution was removed. The plate was then rinsed three times with 300 μl per well of TBST. Patient plasma samples diluted 100-fold with ELISA buffer (TBST containing 10 mg/ml of BSA) were added in a total volume of 50 μl per well and incubated for 1 h at RT. After the plasma was rinsed six times with 200 μl per well of TBST, 4 μg/ml of LigA/GST or 2 μg/ml of LigA/GST in ELISA buffer was added in a total volume of 50 μl per well and incubated for 1.5 h at RT. The antigen solution was then rinsed out as above, and rabbit anti-GST IgG solution (Santa Cruz) diluted at 200-fold with ELISA buffer was added and incubated for 1 h at RT. The antibody solution was then rinsed out as above, then replaced with 50 μl per well of peroxidase-conjugated goat anti-rabbit IgG solution (Bio-Rad) diluted 5000-fold with ELISA buffer and incubated for 1 h at RT. The goat anti-rabbit IgG solution was then rinsed out as above. Finally, 50 μl of *o*-phenylenediamine dihydrochloride solution (OPD tablet (Sigma) in 6 ml distilled, deionized water containing 0.02% hydrogen peroxide) was added and settled for 5 min, and the reaction was stopped by adding 50 μl per well of 1 M sulfuric acid solution. Absorbance at 492 nm of each well was quantitated in a microtiter-plate reader.

### Whole cell-based IgM ELISA (Patoc-IgM ELISA)

The *L*. *biflexa* serovar Patoc antigen coating plate was prepared according to the WHO guidance [5]. The plate was rinsed six times with 200 μl per well of distilled water, and then blocked with 200 μl per well of 20 mg/ml of BSA in TBST for 1.5 h at RT. The plate was then rinsed three times with 300 μl per well of TBST. Patient plasma samples diluted 400-fold with ELISA buffer were added in a total volume of 50 μl per well and incubated for 1.5 h at RT. The plasma was then rinsed six times with 200 μl per well of TBST, then replaced with 50 μl per well of peroxidase-conjugated goat anti-human IgM solution (QED Bioscience) diluted 5000-fold with ELISA buffer and then incubated for 1 h at RT. The goat anti-human IgM solution was then rinsed out as above, and the antigen-bound IgM was detected as describe above.

### Sample size calculation

The sample size was calculated according to the method developed by Flahault *et al* [26]. For an expected sensitivity of 70% with 0.95 probability that the minimum acceptable 95% lower confidence limit of 55%, a sample size of 114 cases was required. For an expected specificity of 98% with 0.95 probability that the minimum acceptable 95% lower confidence limit of 90%, a sample size of 89 controls was required. We therefore aimed to recruit 120 laboratory confirmed cases and 100 healthy controls.

### Data analyses

The Standards for Reporting of Diagnostic Accuracy (STARD) reporting guidelines were followed [27]. A case was defined as laboratory confirmed if 1) culture was positive, 2) specific antibodies were detected with seroconversion or at least a 4-folds increase in reciprocal MAT titer between paired samples or with a reciprocal MAT titer of > = 400 in at least 1 plasma sample, or 3) Lepto-*rrs* LAMP was positive for plasma or urine sample. A case was defined as severe if s/he had acute renal failure (blood urea nitrogen >50 mg/dL, creatinine >5 mg/dL, or required renal dialysis), liver dysfunction (AST >100 IU/dL, ALT >100 IU/dL, or presented with jaundice), pulmonary hemorrhage, or died during hospitalization.

Clinical characteristics were compared between groups using chi-square tests or Fisher’s exact tests for categorical variables and t-tests or Mann-Whitney U test for numerical variables. The ELISA optical density (OD) values were compared between the laboratory-confirmed cases and the blood donor controls using receiver operating characteristic (ROC) analyses. The sensitivities and specificities, with 95% confidence intervals, of each ELISA test was calculated using the mean+3 standard deviation (SD) value of blood donor controls as the cut-off limit for defining a positive result [28].

### Ethics

This study was approved by the Research and Ethics Review Committee of the San Lazaro Hospital and the Institutional Review Board of the Institute of Tropical Medicine, Nagasaki University, Nagasaki, Japan. Informed written consent was obtained from all participants.

## Results

### Overall number of enrolled cases

**Fig 1:**
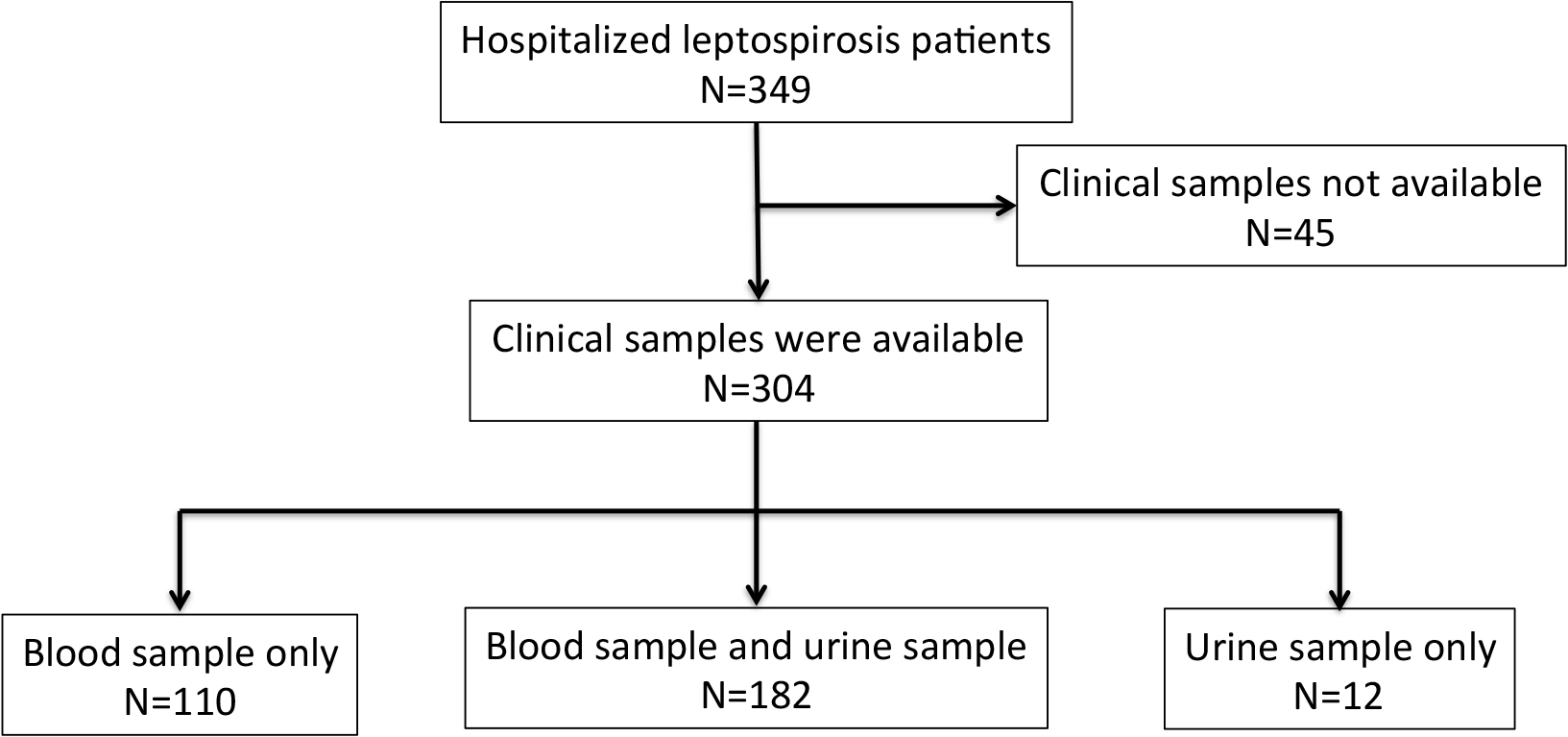
Study enrollment and sample collection.

In total, 349 cases hospitalized with clinically suspected leptospirosis were approached but clinical samples could not be collected from 45 patients during the peak of 2013 outbreak because of overwhelming workload (Fig 1). Consequently 304 patients with the age of 28 years (range 7–67; interquartile range 20–40) were successfully enrolled for the analysis. The demographic and clinical features of the patients are shown in the Tables 1 and S1. Men were predominant (89.1%) and the majority of patients were poor residents of slum areas and had an outside occupation. There were 167/304 (54.9%) patients with severe disease and 12/304 (4.0%) patients died; male had severe disease more frequently than female (57.8% for male and 30.3% for female, p = 0.003) but the gender difference was not observed for mortality (4.4% and 0%, p = 0.2).

**Table 1:** Demographic characteristics of enrolled cases by laboratory confirmation status.

	Total, n = 304		Laboratory confirmed by MAT/culture/LAMP					Laboratory confirmed by LAMP/LigA				
			Confirmed, n = 167		Not confirmed, n = 137		p value	Confirmed, n = 192		Not confirmed, n = 112		p value
	N	%	N	%	N	%		N	%	N	%	
Male gender	270/303	89	158/167	95	112/137	82	<0.01*	178/192	93	92/111	83	<0.01*
Age (median, years old)	28	NA	28	NA	28	NA	0.55	28	NA	29	NA	0.54
Resident in Metro Manila	195/231	84	101/127	80	94/104	90	0.02*	119/145	82	76/86	88	0.14
Pre-hospital antibiotics used	78/219	36	40/127	31	38/92	41	0.09	44/140	31	34/79	43	0.06

* Statistically significant; p<0.05.

### Results of laboratory tests

The 1^st^ blood samples were collected from 292 cases (83.7%) on admission, the 2^nd^ blood samples were collected from 164 cases (47.0%) on discharge and paired 1^st^ and 2^nd^ samples were collected from 162 cases (53.3%). The median duration from onset to the 1^st^ sample collection and the 2^nd^ sample collection varied widely: 6.5 (range 1–39; IQR 2–19) days and 11.2 (range 3–45; IQR 4–27) days, respectively. Urine samples were collected from 195 cases (55.9%). Among the 304 cases tested for MAT and LAMP, 134 (44.1%) were positive by MAT, 58 (19.1%) were positive by LAMP in either plasma or urine or both, and 25 (8.2%) were positive by both MAT and LAMP. LAMP positive plasma samples were found only among samples collected at 9 days or earlier after the disease onset, whereas LAMP positive urine samples were seen throughout (S1 Fig). Culture was performed in 114 cases and five (4.4%) cases were positive; all culture positive cases were positive by LAMP. Taken together, 167 (54.9%) cases were confirmed as leptospirosis based on the results of culture, LAMP and MAT. The most reactive serogroup by MAT was Bataviae (n = 51, 38.1%), followed by Javanica (n = 28, 20.9%), Tarassovi (n = 26, 19.4%), Pyrogenes (n = 20, 14.9%), Sejroe (n = 18, 13.4%), and Grippotyphosa (n = 14, 10.4%). No samples were reactive with Australis, Autumnalis, and Pomona.

### Sensitivities and specificities of ELISAs

The sensitivity of the LigA-IgM ELISA and Patoc-IgM ELISA were calculated using the 167 laboratory confirmed cases by culture, LAMP and MAT as the reference standard. The cut-off OD value (ie. the mean+3SD value of blood donor controls) for the LigA-IgM ELISA and Patoc-IgM ELISA were 0.062 and 0.691, respectively. The sensitivity of LigA-IgM ELISA was significantly higher than that of Patoc-IgM ELISA in the 1st samples on admission: 69.5% (95% confidence interval 60.4–75.1) vs. 54.3% (95% CI 48.3–60.3), respectively; p<0.01. The sensitivities were not significantly different in the 2nd samples taken at the time of discharge: 93.8 vs. 92.0; p = 0.81. Using the blood donor samples as negative control, the specificity was calculated and found high in both assays, 98% (95%CI 96.0–100.0) and 97% (95%CI 94.6–99.5), respectively. The AUC of the ROC curve for the LigA-IgM ELISA was significantly higher than that of Patoc-IgM ELISA in the 1^st^ samples (0.90 vs. 0.82; p<0.01), but this difference was not observed in the 2^nd^ samples (0.97 vs. 0.99; p = 0.18) (S2 Fig).

**Fig 2:**
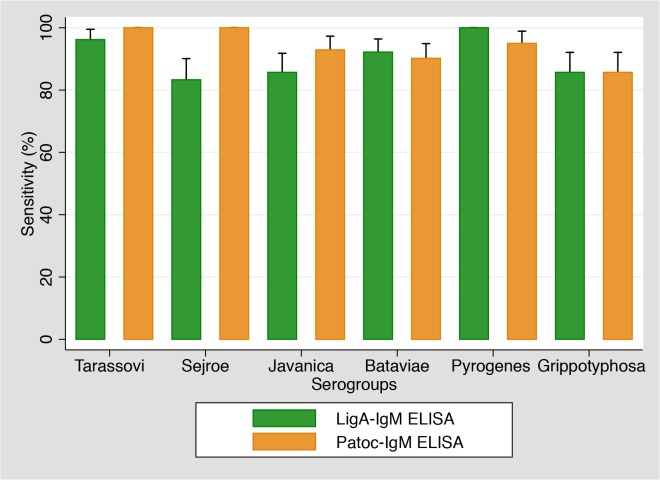
Comparison of sensitivity between serovars (N = 134). Vertical bars indicate 95% confidence intervals.

When sensitivity of LigA-IgM ELISA and Patoc-IgM ELISA was compared according to MAT-positive serogroups (Fig 2), the sensitivity of LigA-IgM ELISA against serogroups Tarassovi, Sejroe, and Javanica were found to be lower than Patoc-IgM ELISA, though the differences were not statistically significant.

### Clinical characteristics of patients according to laboratory confirmation

The demographic and clinical characteristics at the time of admission of the whole 304 cases clinically suspected of leptospirosis were compared, according to the laboratory results (Tables 1 and S1). When compared with clinically suspected but laboratory unconfirmed cases, the laboratory confirmed cases by culture, LAMP and MAT were significantly more likely to be male (94.6% vs. 78.1%; p<0.01). Hemoptysis, jaundice, calf pain, neutrophilia, thrombocytopenia, and renal dysfunction were significantly more common in the laboratory confirmed cases.

Of the 158 MAT-negative cases, 53 (33.5%) were positive by the LigA-IgM ELISA while 28 (17.7%) were positive by the Patoc-IgM ELISA while 12 (9.0%) of the 134 MAT-positive cases were negative by the LigA-IgM ELISA and 10 (7.4%) were negative by the Patoc-IgM ELISA. If the laboratory confirmed cases were defined as those positive by LAMP and LigA-IgM ELISA, the clinical characteristics of cases were more typical of the classic descriptions of leptospirosis when compared with those defined by culture, LAMP and MAT. Cases were clearly associated with jaundice, calf pain, neutrophilia, thrombocytopenia, and renal dysfunction, and there were further associates with myalgia and dyspnea, hypotension, a tendency to have anuria, and a coagulation disorder.

### Association between the LAMP and the LigA antibody level

Leptospiral DNA was detected both in plasma and urine samples: 45 in plasma samples, 22 in urine samples, and 9 in both plasma and urine samples (S1 Fig). The mean (SD) duration of disease was significantly shorter among plasma LAMP positive cases than urine-only positive cases (5.9±1.7 days vs. 9.2±3.3 days; p < 0.01). The mean (SD) LigA-IgM antibody level was lower among plasma LAMP positive cases than plasma LAMP negative cases (0.023±0.511 vs. 0.589±0.587; p = 0.10). LAMP detected leptospiral DNA in 29 LigA negative cases. Intriguingly in the 146 cases that were both plasma and urine LAMP negative the level of LigA antibody was high despite the sample being taken a relatively short time after symptom onset (S3 Fig).

### Sensitivity and day of illness

**Fig 3:**
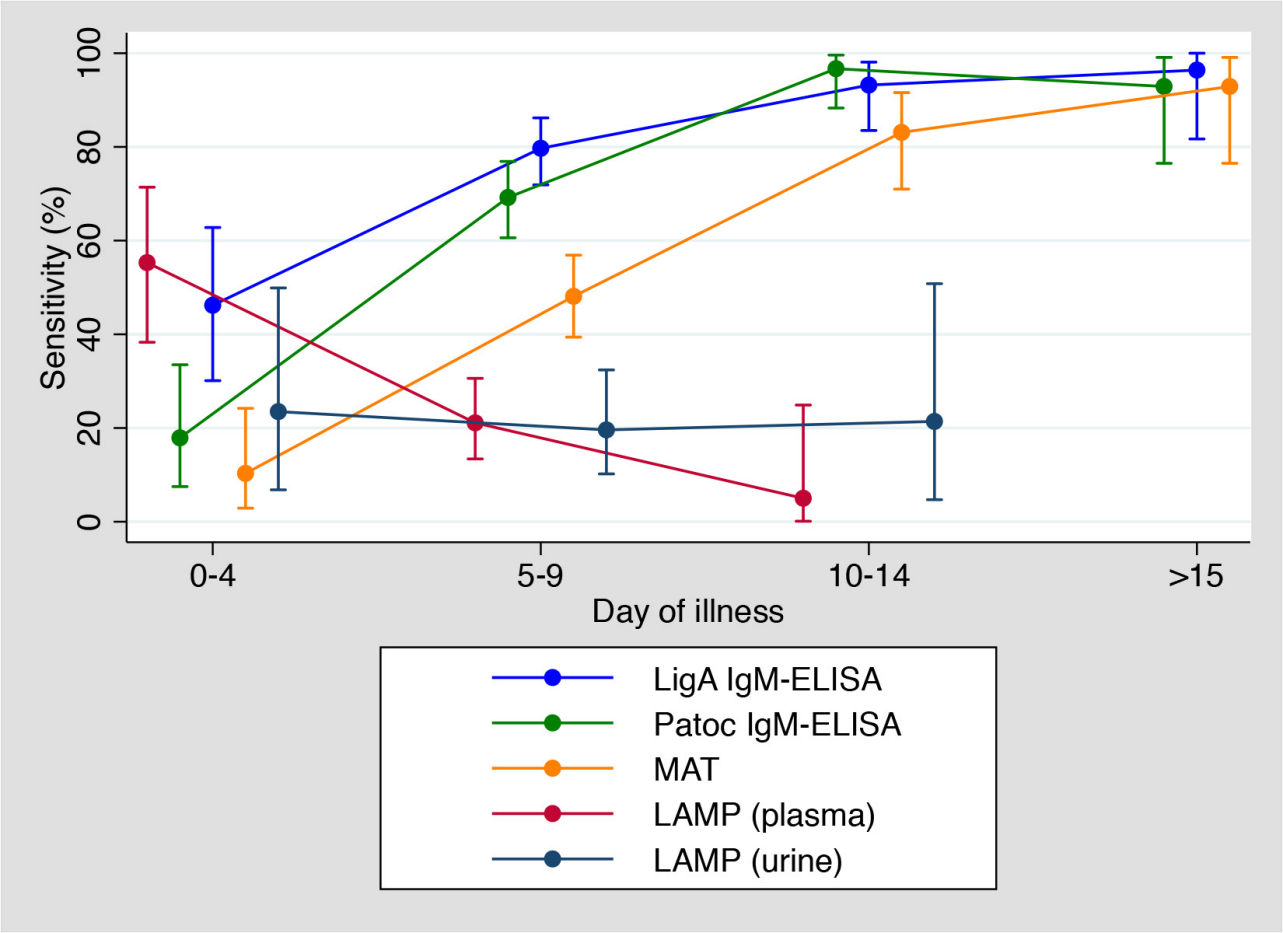
Comparison of sensitivity of diagnostic examination and day of illness among laboratory confirmed cases (N = 153). This figure included pair serum results of MAT and LigA / Patoc-based IgM ELISA. 11 plasma samples and 12 urine samples were not included in this analysis due to a lack of information. Vertical bars indicate 95% confidence intervals.

The sensitivities of each of the diagnostic methods were plotted in relation to the duration from disease onset (Fig 3). In the early phase of disease (0–4 days from onset), LAMP was the most sensitive diagnostic method. As the disease progressed, LAMP sensitivity decreased, whereas the ELISA sensitivities increased. The LigA-IgM ELISA showed a higher sensitivity in the early phase than the other serological tests. The MAT sensitivity was lower than the sensitivity of both ELISAs throughout.

## Discussion

This is the first hospital-based prospective study, evaluating the diagnostic accuracy of the LigA-IgM ELISA for leptospirosis. The study was conducted in a busy government referral hospital serving the poorest people in a highly-endemic area of Metro Manila. There were several large outbreaks during the study period. Our results demonstrated that LigA-IgM ELISA has a high clinical value because a) it had a higher sensitivity than the Patoc-IgM ELISA, especially in the first 10 days after onset, and b) leptospirosis was diagnosed in a substantial number of patients, whose diagnosis would have been missed by the conventional laboratory confirmation with MAT.

### Reason for the better performance of LigA assay than the whole cell-based assay

The whole cell-based serology assays are commercially available, but previous studies have demonstrated that they have a low sensitivity, especially in the early phase of leptospiral infection and low specificity [12,29]. The recombinant protein-based serology assay is thought to achieve a higher sensitivity and specificity because of the higher concentrations of immunogenic antigens and specific antigenic moieties [18]. Furthermore, antibodies detected by the whole cell-based assay persist for years after leptospiral infection and this causes a major problem of low specificity in an endemic area. Croda reported that 29% of sera from patients with previous leptospirosis remained positive by whole-*Leptospira*-based IgM ELISA for 4–6 years after infection [30].To reduce this problem, in our study, we determined the cut-off OD for the whole cell-based serology assay by using the local blood donors as negative controls. If the cut-off OD had been determined by using non-leptospirosis Japanese febrile patients as negative controls (n = 15), the cut-off OD was lower, resulting in a higher sensitivity (73.2% in the 1st samples) but a lower specificity (78.0% in the 1st samples). The cut-off OD for the LigA-IgM ELISA did not significantly differ depending on the negative control samples used.

### Previously published studies of LigA-IgM ELISA

Several studies have evaluated the performance of LigA-IgM ELISA. The sensitivity has varied depending on sample population studied, the duration from the disease onset and also the definition of the composite reference standard. For an appropriate evaluation, we need to use several diagnostic assays to provide an adequate composite reference standard and a sufficiently large sample size. Very few studies fulfilled such criteria. Srimanote et al, reported a sensitivity of LigA-IgM ELISA was over 94% in both acute and convalescent phase when the cases were defined by a positive MAT result, but their sample size was very small (n = 46) and detection of pathogen was attempted only by culture [28]. Chen et al., reported the sensitivity of recombinant protein-based ELISA using LipL32, LipL41 and LigA antigens to be as low from 62 to 65%, although it could be increased to 90% by combing more than 2 antigens [20]. Very recently Kanagavel et al., compared the LigA-IgM ELISA and whole cell-based IgM ELISA in a study with a sufficient sample size (n = 140) and demonstrated that the sensitivity and the specificity of the LigA-IgM ELISA was higher than that of the whole cell-based ELISA [31]. These studies only evaluated the sensitivity using MAT positive samples referred for reference laboratory testing of unknown clinical background. There have been no previous studies evaluating the performance of the assay in a real clinical setting.

### Influence of serogroup on ELISA results

Our results suggested slight differences in sensitivity of the LigA-IgM ELISA depending on the infecting serogroups. Although the differences were not statistically significant, the sensitivity of LigA-IgM ELISA tended to be lower against *L*. *borgpetersenii* (serogroups Sejroe, Tarassovi and Javanica) but higher against *L*. *interrogans* (serogroups Bataviae, Pyrogenes and Grippotyphosa) when compared with the whole cell-based ELISA. A recent study suggested that *L*. *interrogans* expresses genes of both LigA and LigB but *L*. *borgpetersenii* has only LigB [32,33]. A lack of LigA in *L*. *borgpetersenii* may explain the lower sensitivity. It is plausible that, although the LigB molecule has the same repeated immunoglobulin-like domains where antibodies bind to, the C-terminal 80 kDa domain of LigB might mask the antibody epitope regions [34].

### Practical laboratory definition for leptospirosis in clinical settings

Culture and MAT is still regarded as the composite reference standard for leptospirosis referral laboratory confirmation. This definition has a number of limitations in the clinical setting. Both tests demand well-established laboratory where specific culture media is available for leptospirosis and the MAT assay needs an experienced laboratory technician with several *Leptospira* serovar strains alive. Culture requires up to 13 weeks to have results and can be rendered negative by antibiotic pre-exposure. To overcome these limitations, in this study, we used a LAMP assay that does not demand sophisticated laboratory equipment and that gives a result within 2 hours with almost equivalent sensitivity to real-time PCR. When we applied a new definition by LAMP and LigA-IgM ELISA and compared with culture/LAMP/MAT, we identified a patient population whose clinical symptoms and signs were more compatible with leptospirosis than culture/LAMP/MAT. We propose that the LAMP/LigA ELISA combination is practical and reasonable for laboratory confirmation in the clinical setting.

### Limitation

There are several limitations in this study. The study was conducted in a busy government hospital, many patients were discharged within seven days after admission and did not return to the clinic, thus convalescent samples were often not available at the optimum time of at least two weeks apart. This is a reflection of a real clinical setting. The majority of our patients were males reflecting the high incidence of leptospirosis among males in our setting [3]. However, this imbalance did not affect our findings; the sensitivity and specificity of LigA-IgM ELISA and Patoc-IgM ELISA did not differ between the sexes. Most laboratory tests were conducted in Japan, and some LAMP results might have been affected by improper condition of sample transportation. Finally, the lack of a satisfactory laboratory composite reference standard is a limitation in defining the diagnostic accuracy of new diagnostic tests in leptospirosis.

In summary, the findings of this study indicate that LigA-IgM ELISA can be a good diagnostic tool with high sensitivity especially in early phase of illness. We propose that a combination of molecular and serology assays can improve diagnosis and help timely initiation of antibiotics to prevent severe outcomes of leptospirosis in an endemic area.

## Supporting Information

S1 Checklist STARD Checklist. (DOC)

S1 Table Clinical characteristics of enrolled cases by laboratory confirmation status. * Statistically significant; p<0.05.
(DOC)

S1 Fig Distribution of the results of Lepto-*rrs* LAMP in plasma and/or urine of the confirmed cases (N = 36). P: LAMP of plasma; U: LAMP of urine.
(TIFF)

S2 Fig ROC curves of LigA-based IgM-ELISA and Patoc-based IgM-ELISA among confirmed leptospirosis cases (N = 167). a. LigA-based IgM ELISA in 1^st^ samples; b. LigA-based IgM ELISA in 2^nd^ samples; c. Patoc-based IgM ELISA in 1^st^ samples; d. Patoc-based IgM ELISA in 2^nd^ samples. The area under ROC curve of LigA-based and Patoc-based IgM ELISA were 0.90 and 0.82, respectively in 1^st^ samples (p<0.01) and 0.97 and 0.99, respectively in 2^nd^ samples (p = 0.18).
(TIF)

S3 Fig LigA IgM-ELISA OD values by LAMP results. P: LAMP of plasma; U: LAMP of urine. Each dot represents single plasma sample. Horizontal lines indicate median values.
(TIF)
