# Supplementary material for: Diagnostic Accuracy of Recombinant Immunoglobulin-like Protein A-Based IgM ELISA for the Early Diagnosis of Leptospirosis in the Philippines
Source: PLoS Negl Trop Dis. 2015 Jun 25;9(6):e0003879. doi: 10.1371/journal.pntd.0003879 (PMC4482399; doi:10.1371/journal.pntd.0003879)
Supplement: S1 Table — * Statistically significant; p<0.05. (DOC) [file pntd.0003879.s002.doc]

|  | Total, n=304 | | Laboratory confirmed by LAMP/LigA | | | | |
| --- | --- | --- | --- | --- | --- | --- | --- |
|  |  |  | Confirmed, n=192 | | Not confirmed, n=112 | | p value |
|  | N | % | N | % | N | % |  |
| Demography |  |  |  |  |  |  |  |
| Male gender | 270/303 | 89 | 178/192 | 93 | 92/111 | 83 | <0.01* |
| Age (median, years old) | 28/304 | NA | 28 | NA | 29 | NA | 0.54 |
| Resident in Metro Manila | 195/231 | 84 | 119/145 | 82 | 76/86 | 88 | 0.14 |
| Pre-hospital antibiotics used | 78/219 | 36 | 44/140 | 31 | 34/79 | 43 | 0.06 |
| Symptoms |  |  |  |  |  |  |  |
| Pyrexia | 282/284 | 99 | 181/183 | 99 | 101/101 | 100 | 0.41 |
| Headache | 182/284 | 64 | 111/183 | 61 | 71/101 | 70 | 0.07 |
| Myalgia | 170/284 | 60 | 120/183 | 66 | 50/101 | 50 | <0.01* |
| Cough | 90/284 | 32 | 54/183 | 30 | 36/101 | 36 | 0.18 |
| Dyspnea | 72/284 | 25 | 55/183 | 30 | 17/101 | 17 | <0.01* |
| Hemoptysis | 17/284 | 6 | 15/183 | 8 | 2/101 | 2 | 0.03* |
| Jaundice | 75/284 | 26 | 57/183 | 31 | 18/101 | 18 | 0.01* |
| Malaise | 155/284 | 55 | 96/183 | 52 | 59/101 | 58 | 0.20 |
| Vomiting/nausea | 189/284 | 67 | 128/183 | 70 | 61/101 | 60 | 0.07 |
| Diarrhea | 87/284 | 31 | 61/183 | 33 | 26/101 | 26 | 0.12 |
| Calf pain | 138/284 | 49 | 103/137 | 75 | 35/77 | 45 | <0.01* |
| Conjuctival suffusion | 164/284 | 58 | 112/183 | 61 | 52/101 | 51 | 0.07 |
| Abdominal pain | 170/284 | 60 | 113/183 | 62 | 57/101 | 56 | 0.23 |
| Oliguria | 101/284 | 36 | 67/183 | 37 | 34/101 | 34 | 0.36 |
| Anuria | 18/284 | 6 | 15/183 | 8 | 3/101 | 3 | 0.07 |
| Tea color urine | 60/284 | 21 | 35/183 | 19 | 25/101 | 25 | 0.17 |
| Dehydration | 45/284 | 16 | 33/183 | 18 | 12/101 | 12 | 0.12 |
| Dizziness | 59/284 | 21 | 34/183 | 19 | 25/101 | 25 | 0.14 |
| Vital signs |  |  |  |  |  |  |  |
| Hyperthermia, ≥38^o^C | 97/283 | 34 | 55/181 | 30 | 42/102 | 41 | 0.045* |
| Hypotension, <100 mmHg | 101/281 | 36 | 74/179 | 41 | 27/102 | 26 | <0.01* |
| Tachycardia, >100 beats/min | 100/283 | 35 | 65/181 | 36 | 35/102 | 34 | 0.45 |
| Tachypnea, >20 breaths/min | 230/279 | 82 | 151/179 | 84 | 79/100 | 79 | 0.17 |
| Laboratory |  |  |  |  |  |  |  |
| Neutrophilia, >8×10^9^cells/L | 118/272 | 43 | 89/173 | 51 | 29/99 | 29 | <0.01* |
| Anemia, <10.0g/dL | 23/277 | 8 | 18/177 | 10 | 5/100 | 5 | 0.10 |
| Thrombocytopenia, <10×10^4^cells/L | 70/273 | 26 | 53/174 | 30 | 17/99 | 17 | 0.01* |
| Liver dysfunction (AST>37 and/or ALT>41) | 117/158 | 74 | 78/105 | 74 | 39/53 | 74 | 0.54 |
| Renal dysfunction (BUN>20.16 and/or Cr>1.29) | 196/262 | 75 | 159/171 | 93 | 37/91 | 41 | <0.01* |
| Coagulation disorder (PT-INR>1.0) | 66/96 | 69 | 43/68 | 63 | 23/28 | 82 | 0.06 |
| Urynalysis abnormal | 134/210 | 64 | 87/130 | 67 | 47/80 | 59 | 0.15 |
| Chest X-ray abnormal | 66/153 | 43 | 45/103 | 44 | 21/50 | 42 | 0.49 |
