# Supplementary figures and images for: Diagnostic Accuracy of Recombinant Immunoglobulin-like Protein A-Based IgM ELISA for the Early Diagnosis of Leptospirosis in the Philippines
Source: PLoS Negl Trop Dis. 2015 Jun 25;9(6):e0003879. doi: 10.1371/journal.pntd.0003879 (PMC4482399; doi:10.1371/journal.pntd.0003879)

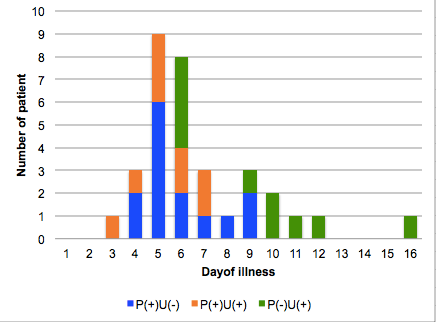

Supplement: S1 Fig — P: LAMP of plasma; U: LAMP of urine. (TIFF) [file pntd.0003879.s003.tiff]

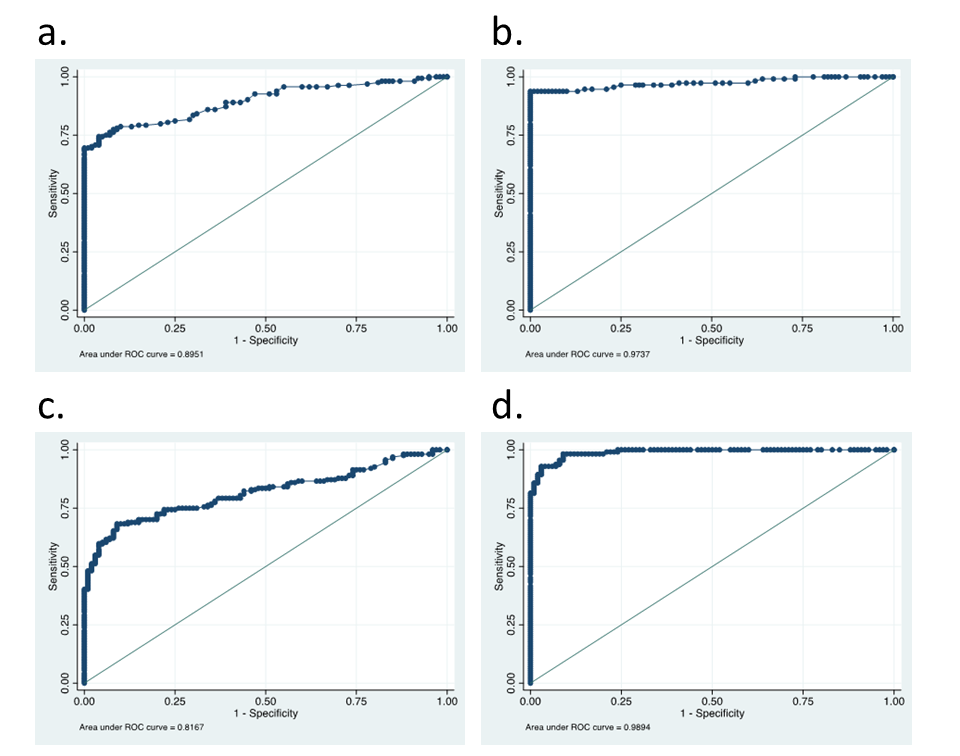

Supplement: S2 Fig — a. LigA-based IgM ELISA in 1st samples; b. LigA-based IgM ELISA in 2nd samples; c. Patoc-based IgM ELISA in 1st samples; d. Patoc-based IgM ELISA in 2nd samples. The area under ROC curve of LigA-based and Patoc-based IgM ELISA were 0.90 and 0.82, respectively in 1st samples (p<0.01) and 0.97 and 0.99, respectively in 2nd samples (p = 0.18). (TIF) [file pntd.0003879.s004.tif]

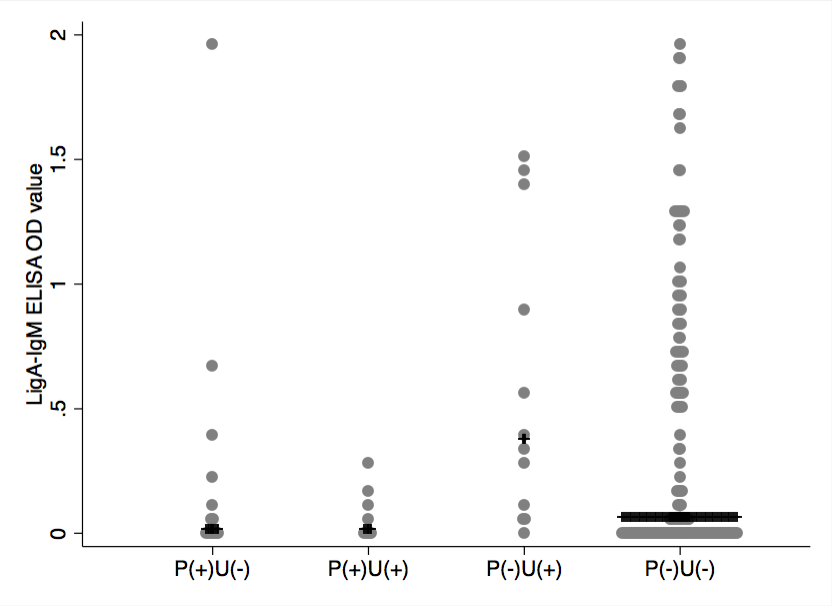

Supplement: S3 Fig — P: LAMP of plasma; U: LAMP of urine. Each dot represents single plasma sample. Horizontal lines indicate median values. (TIF) [file pntd.0003879.s005.tif]
